# Alterations in amino acid status in cats with feline dysautonomia

**DOI:** 10.1371/journal.pone.0174346

**Published:** 2017-03-23

**Authors:** Bruce C. McGorum, Herb W. Symonds, Clare Knottenbelt, Tom A. Cave, Susan J. MacDonald, Joanna Stratton, Irene Leon, Judith A. Turner, R. Scott Pirie

**Affiliations:** 1 Royal (Dick) School of Veterinary Studies and The Roslin Institute, University of Edinburgh, Roslin, Midlothian, United Kingdom; 2 Freelance, Harrogate, North Yorkshire, United Kingdom; 3 School of Veterinary Medicine, University of Glasgow, Glasgow, Strathclyde, United Kingdom; 4 Cave Vet Specialists, Wellington, Somerset, United Kingdom; 5 Fera Science Limited, Sand Hutton, York, United Kingdom; University of Minnesota, UNITED STATES

## Abstract

Feline dysautonomia (FD) is a multiple system neuropathy of unknown aetiology. An apparently identical disease occurs in horses (equine grass sickness, EGS), dogs, rabbits, hares, sheep, alpacas and llamas. Horses with acute EGS have a marked reduction in plasma concentrations of the sulphur amino acids (SAA) cyst(e)ine and methionine, which may reflect exposure to a neurotoxic xenobiotic. The aim of this study was to determine whether FD cats have alterations in amino acid profiles similar to those of EGS horses. Amino acids were quantified in plasma/serum from 14 FD cats, 5 healthy in-contact cats which shared housing and diet with the FD cats, and 6 healthy control cats which were housed separately from FD cats and which received a different diet. The adequacy of amino acids in the cats’ diet was assessed by determining the amino acid content of tinned and dry pelleted foods collected immediately after occurrences of FD. Compared with controls, FD cats had increased concentrations of many essential amino acids, with the exception of methionine which was significantly reduced, and reductions in most non-essential amino acids. In-contact cats also had inadequate methionine status. Artefactual loss of cysteine during analysis precluded assessment of the cyst(e)ine status. Food analysis indicated that the low methionine status was unlikely to be attributable to dietary inadequacy of methionine or cystine. Multi-mycotoxin screening identified low concentrations of several mycotoxins in dry food from all 3 premises. While this indicates fungal contamination of the food, none of these mycotoxins appears to induce the specific clinico-pathologic features which characterise FD and equivalent multiple system neuropathies in other species. Instead, we hypothesise that ingestion of another, as yet unidentified, dietary neurotoxic mycotoxin or xenobiotic, may cause both the characteristic disease pathology and the plasma SAA depletion.

## Introduction

Feline dysautonomia (FD) is a multiple system neuropathy first reported in Scotland in 1982 [1]. It is characterised by chromatolysis, degeneration and loss of enteric neurons, peripheral and central autonomic neurons, neurons in specific brain stem nuclei and spinal cord somatic efferent lower motor neurons [2,3]. The predominant clinical features of FD are attributable to paralysis of the entire gastrointestinal tract caused by severe enteric neuropathy [3]. Striking similarities in the clinico-pathological features of FD with multiple system neuropathies reported in horses (equine grass sickness; EGS), dogs (canine dysautonomia), hares (leporine dysautonomia), rabbits, alpacas, llamas and sheep (abomasal emptying defect) suggest these represent a specific disease entity with a common aetiology [4]. The authors are unaware of a human neurodegenerative disorder which shares the exquisitely localised neuroanatomic lesions of these animal diseases. The aetiology of these animal multiple system neuropathies is unknown, although some evidence supports an association with *Clostridium botulinum* type C/D [2,5–7]. There has been no investigation of the potential role of genetic mutations, such as those which cause familial human dysautonomia [8]. Horses with acute EGS have reductions in plasma concentrations of the sulphur amino acids (SAA) cyst(e)ine and methionine, and perturbations in plasma amino acid profile consistent with severe protein malnutrition [9]. We hypothesise that SAA depletion in EGS may reflect xenobiotic exposure, since SAA are strong nucleophiles that trap, reduce, detoxify and facilitate the excretion of electrophilic compounds and free radicals derived from dietary toxins such as mycotoxins, plant lectins, sesquiterpene lactones and toxic amino acids [10–13]. The aim of this study was to determine whether FD cats have alterations in amino acid profiles similar to those of EGS horses. Amino acids were quantified in plasma/serum from 14 FD cats from 3 separate premises, 5 healthy in-contact cats which shared housing and diet with the FD cats, and 6 healthy control cats which were housed separately from FD cats and which received a different diet. The adequacy of the cats’ dietary amino acid intake was assessed by determining the amino acid content of 2 tinned and 5 dry pelleted foods collected from the 3 premises immediately after occurrences of FD. Multi-mycotoxin screening was performed on dry food from all 3 premises to determine whether FD and altered plasma amino acid status could be associated with ingestion of a dietary neurotoxic mycotoxin.

## Materials and methods

### Sample collection

The study was approved by the School of Veterinary Medicine Ethical Review Committee, University of Edinburgh. Samples were collected from 3 premises shortly after occurrences of FD. Premise A was a closed cat breeding colony in north England where previously published cases of FD had occurred [14,15]. These publications provide detailed information on colony management and epidemiology of previous cases. Samples were collected for the present study when an additional 23 cats developed FD. Heparinised plasma was collected from 13 of these FD cats (median age 11 weeks, range 8 weeks-26 months) and from 5 healthy in-contact cats (age 14 weeks, 8 weeks-36 months) which were housed with the FD cats and given the same food. FD and in-contact cats were fed approximately 114 g/day tinned food in the morning and ad libitum dry pellets in the afternoon (Table 1); samples of these foods were collected for analysis. Heparinised plasma was also collected from 6 healthy control cats (3 males, 3 females; median age 32 weeks, range 11–36 weeks) which were housed separately on premise A. These control cats received a different diet from the FD and in-contact cats (this diet was not analysed). Premise B was a closed non-breeding colony in south Scotland, where 6 of 8 cats developed clinical FD, 3 of which were euthanased on humane grounds. Cats were fed dry pellets from a hopper, tinned food and small amounts of turkey and ham. Tinned food, dry pellets (Table 1) and serum from one FD cat (Birman, female entire, 5 years old) were collected from premise B. Food (Table 1) was collected from Premise C, a single cat household in north Scotland. Blood samples were not available from cats on premise C. Detailed clinical and epidemiological information for the clusters on premises B and C was published [16]. FD was confirmed by demonstrating characteristic gross necropsy findings, and histological evidence of chromatolysis, degeneration and loss of autonomic neurons in the cranial cervical and/or celiacomesenteric ganglia [2,3], for all FD cats from premise A, 2/8 cats from premise B and the cat from premise C.

**Table 1:** Metabolisable energy (ME; kcal/100g), protein and amino acid (g/1000 kcal ME) content of cat foods (n = 7) collected immediately after occurrences of FD. Also shown are [cystine + methionine] and [phenylalanine + tyrosine] concentrations. Recommended minimum nutrient requirements for growing and breeding cats (g/1000 kcal ME) are derived from the FEDIAF (2014) Nutritional Guidelines for Complete and Complementary Pet Food for Cats and Dogs [17].

	Premise A Tinned food	Premise A Dry pellets	Premise B Tinned food	Premise B Dry pellets	Premise C Dry pellets 1	Premise C Dry pellets 2	Premise C Dry pellets 3	Recommended minimum requirement
**ME**	78	333	66	448	346	326	326	-
**Protein**	105	94	121	68	91	94	104	70/75
**Alanine**	6.0	5.8	8.3	4.4	6.6	5.9	6.3	-
**Arginine**	7.8	5.7	12.1	6.3	5.7	7.4	8.0	2.68/2.78
**Aspartic Acid**	8.2	7.2	11.1	6.0	6.1	7.4	7.9	-
**Cystine**	0.5	1.2	1.1	0.9	1.5	1.2	1.2	-
**Glutamic Acid**	12.3	17.5	17.0	10.1	18.9	16.3	16.6	-
**Glycine**	8.5	6.3	12.0	6.0	4.3	7.1	8.1	-
**Histidine**	2.4	2.1	3.2	1.6	2.0	2.3	2.3	0.83
**Isoleucine**	3.5	3.3	4.7	2.9	3.6	3.8	3.8	1.35
**Leucine**	7.2	8.0	9.2	5.3	11.0	8.2	8.2	3.20
**Lysine**	5.9	4.2	8.6	4.1	2.8	4.7	5.0	2.13
**Methionine**	1.8	1.7	2.3	2.1	1.9	1.8	2.1	1.10
**Phenylalanine**	3.8	4.1	5.2	2.8	4.9	4.4	4.3	1.25
**Proline**	5.6	7.0	8.0	4.8	7.5	6.9	6.9	-
**Serine**	3.8	4.1	5.3	3.9	4.6	4.4	4.4	-
**Threonine**	3.6	3.2	5.0	2.9	3.2	3.6	3.6	1.63
**Tyrosine**	2.9	3.0	3.9	2.2	4.0	3.3	3.3	-
**Valine**	5.0	4.2	6.5	3.8	4.3	4.6	4.6	1.60
**Meth + Cys**	2.3	2.8	3.3	3.0	3.5	3.0	3.3	2.20
**Phe + Tyr**	6.8	7.1	9.1	5.0	12.6	7.7	7.5	4.78

Blood was collected from cats within 24 h of the onset of clinical signs, centrifuged at 500g for 10–15 min and plasma/serum stored at -70°C prior to analysis within 2 months. Plasma was not deproteinised prior to freezing. Prior to analysis, dry food was stored in sealed plastic bags at –20°C, while unopened canned food was stored at room temperature.

### Plasma/serum amino acid analyses

Plasma/serum amino acid profiles were determined as previously described [9]. Tryptophan was not detectable by this method due to losses during sample preparation and analysis. Cysteine and cystine were not differentiated by this method, because cysteine was oxidised to the disulphide cystine during analysis. The combined cysteine and cystine concentration was therefore referred to as the cyst(e)ine concentration.

### Amino acid content of food

Amino acids were quantified using acid hydrolysis, with the exception of cysteine and methionine which were quantified using performic acid oxidation to convert the amino acids to the acid stable compounds cysteic acid and methionine sulphone [18]. Protein content was calculated using the Kjeldahl technique [19]. Protein and amino acid content were expressed as g/1000 kcal metabolisable energy (ME), using the manufacturers’ stated ME values for each food. The adequacy of the protein and amino acid content of foods was determined using the FEDIAF Nutritional Guidelines for Complete and Complementary Pet Food for Cats and Dogs (2014) [17] and the National Research Council (NRC) Nutrient Requirements for Dogs and Cats (2016) [20].

### Multi-mycotoxin screening of dry food

Dry cat food from all 3 premises was subjected to multi-mycotoxin screening using published LC-MS/MS methodology [21]. In brief, this involved extraction of samples on a shaker with a mixture of acetonitrile water acetic acid (79 20 1). After centrifugation, an aliquot of the test sample extract was diluted with an equal volume of a mixture of acetonitrile water acetic acid (20 79 1). This was filtered through 0.2μm pore size nylon syringe filter before analysis by UPLC-MS/MS. Two chromatography runs were required to determine all mycotoxins, the majority of compounds were run using a neutral mobile phase gradient. The remaining compounds (fumonisins, ochratoxin A, citrinin and alternaria toxins) were analysed using acidic conditions. Samples were analysed as received and spiked at the Level of Interest, this was used to determine individual reporting limits. The 72 mycotoxins tested for and their respective reporting limits are listed in S1 Table.

### Statistical analysis

As the concentrations of all serum amino acids for the single FD cat from premise B were within the ranges for plasma amino acids for the 13 FD cats from premise A, data for all 14 FD cats were pooled for analysis. As data were non-normally distributed, the Mann Whitney Test was used for inter-group comparisons. For statistical analysis, values below the appropriate detection limits (<5uM or <10uM) were considered to be 4.9uM and 9.9uM, respectively. As the inter-group comparison of plasma amino acid concentrations involved numerous (n = 63) statistical comparisons, a significance level of p<0.01 was used to reduce the probability of significant differences occurring due to chance.

## Results

Plasma/serum amino acid data are summarised in Table 2, with data for individual cats being given in S2 Table.

**Table 2:** Concentrations of amino acids (μmol/L) in plasma/serum from FD (n = 14), in-contact (n = 5) and control cats (n = 6).

	FD	IN-CONTACTS	CONTROLS
**Alanine**	450 (249–894)	725 (399–957)	486 (278–694)
**Arginine**	135 (26–246)	101 (54–182)	98 (50–115)
**Aspartate**	37 (19–97) ^c^	92 (62–116) ^c^	65 (33–89)
**Cyst(e)ine**	11 (<5–35)	15 (<10–22)	<10 (<10-<10)
**Glutamate**	98 (46–179) ^b^	478 (298–532) ^b^^c^	109 (19–124) ^c^
**Glutamine**	489 (346–912)^b^	437 (266–550) ^c^	843 (729–1323) ^b^^c^
**GLU + GLN**	576 (444–958)	848 (744–996)	949 (748–1435)
**Glycine**	398 (244–902)	487 (349–626)	606 (360–880)
**Histidine**	107 (59–169)	101 (69–143)	105 (71–167)
**Isoleucine**	98 (53–137) ^a^	73 (40–87)	38 (24–59) ^a^
**Leucine**	180 (133–230) ^b^	163 (85–234)	108 (53–156) ^b^
**Lysine**	201 (140–321) ^a^^b^	117 (62–138) ^b^	94 (64–120) ^a^
**Methionine**	49 (33–90) ^b^	11 (<10–64) ^c^	77 (65–122) ^b^^c^
**Ornithine**	69 (24–258) ^b^^c^	36 (33–53) ^b^	34 (29–39) ^c^
**Phenylalanine**	73 (58–107) ^b^	53 (39–60) ^b^	61 (49–80)
**Serine**	233 (145–432)	280 (236–352)	238 (193–650)
**Taurine**	204 (67–489)	211 (116–276)	115 (60–179)
**Threonine**	172 (126–294) ^c^	157 (133–219)	121 (105–155) ^c^
**Tyrosine**	42 (28–75)	28 (25–63)	42 (27–59)
**Valine**	287 (225–369) ^a^^b^	151 (128–253) ^b^	121 (83–159) ^a^

^a^ significant difference between pairs (p<0.001)

^b^ significant difference between pairs (p<0.005)

^c^ significant difference between pairs (p<0.01).

GLU + GLN—sum of glutamate and glutamine concentrations.

Comparison of data from the three groups of cats facilitates evaluation of some of the potential factors which could influence amino acid status. Differences in amino acid status between FD and control cats could reflect differences in diet or management, direct effects of the putative causal neurotoxin or the numerous metabolic perturbations resulting from clinical FD including anorexia, tissue catabolism and gastrointestinal ileus. Differences between FD and in-contact cats could reflect differences in magnitude of exposure to the putative neurotoxin or the secondary clinical consequences of FD. Differences between in-contact and control cats could reflect differences in diet or management, or the effects of potential sub-clinical exposure of the in-contact cats to the putative neurotoxin which causes FD.

Plasma/serum amino acid concentrations for control cats were generally similar to those reported previously [22–24], except for artefactual alterations in cyst(e)ine and glutamate concentrations. Cyst(e)ine concentrations were lower than previously reported (75 ± 3 μmol/L [22]; 75 ± 2 μmol/L [23]; >40 μmol/L [25]). Cyst(e)ine was undetectable in 5 FD and 2 in-contact cats. Most cats had higher glutamate concentrations than previously reported (72 ± 19 μmol/L [23]; 32 μmol/L [25]).

Compared with controls, FD cats had significantly increased concentrations of isoleucine (p<0.001), leucine (p<0.005), lysine (p<0.001), ornithine, threonine (p<0.01) and valine (p<0.001), and significantly decreased concentrations of glutamine and methionine (p<0.005). Compared with in-contacts, FD cats had significantly increased concentrations of lysine, ornithine and valine (p<0.005) and significantly decreased concentrations of aspartate (p<0.01) and glutamate (p<0.005). Compared with controls, in-contact cats had significantly increased concentrations of glutamate, and significantly reduced concentrations of glutamine and methionine (p<0.01).

Methionine concentrations for FD and in-contact cats were markedly lower than reported previously for healthy cats (range 65 ± 12 μmol/L [22]; 66 ± 8 μmol/L [23]; 75 ± 34 μmol/L [26]; 101.1 ± 8.85 μmol/L [27]). Methionine was limiting (<30 μmol/L [20]) in 3 in-contacts, one of which had an undetectable methionine concentration (S2 Table). Methionine concentrations of these 3 in-contact cats approximated those of kittens fed diets lacking in methionine (11 μmol/L [20]). Tyrosine concentrations were limiting (<35 μmol/L [20]) in 5 FD, 2 in-contact and 2 control cats (S2 Table). Arginine was limiting (<75 μmol/L [20]) in 4 FD, 2 in-contact and 2 control cats (S2 Table).

Data for food analyses are given in Table 1. The protein and essential amino acid content of all 7 foods exceeded FEDIAF (2014) [17] minimum recommended levels for growing kittens. Tinned food from premise B had an arginine content (12.1 g/1000 kcal ME) exceeding the NRC 2006 [20] recommended upper limit (8.75 g/1000 kcal ME). Dry pellets 1 from premise C had a glutamic acid content (18.9 g/1000 kcal ME) just exceeding the NRC 2006 [20] recommended upper limit (18.8 g/1000 kcal ME).

Dry cat food from all 3 premises contained beauvericin, deoxynivalenol, enniatins (A1, B and B1), fumonisin (B1, B2 and B3), fusarenon X and zearalenone (Table 3). Deoxynivalenol-3-glucoside was also detected in feed from premises 1 and 3 and mycophenolic acid was also detected in feed from premises 2 and 3 (Table 3).

**Table 3:** Concentrations of mycotoxins (μg/kg) in dry food from the 3 premises. Data are concentrations measured in 3 aliquots of each sample.

Mycotoxin	Premise 1			Premise 2			Premise 3		
Beauvericin	600	601	601	557	566	562	524	574	549
Deoxynivalenol	241	232	236	7	11	9	3	5	4
Deoxynivalenol-3-Glucoside	28	26	27	-	-	-	0	11	5
Emodin	-	-	-	30	43	37	72	33	53
Enniatin A1	5	5	5	6	6	6	8	10	9
Enniatin B	35	33	34	19	18	19	19	23	21
Enniatin B1	35	36	36	30	29	30	31	41	36
Fumonisin B1	180	146	163	44	46	45	39	39	39
Fumonisin B2	118	121	119	72	73	73	63	65	64
Fumonisin B3	37	35	36	22	22	22	20	21	21
Fusarenon X	17	15	16	30	29	29	15	20	17
Mycophenolic Acid	-	-	-	18	17	17	16	17	16
Zearalenone	72	79	76	22	20	21	17	15	16

## Discussion

Cats with FD had significant alterations in plasma/serum amino acid status when compared with clinically healthy in-contact and control cats. Compared with controls, FD cats had significantly increased concentrations of many essential amino acids, with the exception of methionine which was significantly reduced, and reductions in most non-essential amino acids. The increase in essential amino acids in FD may reflect increased tissue protein catabolism in response to reduced food intake. In contrast, horses with EGS had reduced plasma concentrations of many essential amino acids, consistent with severe protein malnutrition, and increased concentrations of some non-essential amino acids [9].

Perhaps the most notable finding was low plasma/serum concentrations of methionine in FD and in-contact cats. Indeed methionine was undetectable (<10 μmol/L) in plasma from one in-contact cat, while 2 other in-contact cats had only 11 μmol/L methionine which equates to the plasma methionine concentration of kittens fed diets lacking methionine [20,25]. Methionine is an essential amino acid for cats, although cysteine may supply approximately 50% of this requirement [28]. Many of the FD cats in this study were young growing cats which have a higher SAA requirement than adults [20].

The low concentrations of methionine noted in FD and in-contact cats is consistent the depletion of methionine and cyst(e)ine reported in EGS and healthy in-contact horses [9]. In the present study it was unfortunately not possible to meaningful assess the cats’ cyst(e)ine status because the low plasma/serum cyst(e)ine concentrations noted in all cats were likely artefactual, due to cysteine interaction with sulphydryl groups on plasma proteins and subsequent loss when the protein was precipitated during sample preparation. This loss occurs rapidly in feline plasma/serum that is not deproteinised immediately after collection [29].

While methionine is generally the limiting amino acid for feline diets that are formulated using natural ingredients [20], the low methionine noted in FD and in-contact cats is unlikely to reflect inadequate dietary intake since the methionine, cystine and [methionine + cysteine] content of all foods tested exceeded the minimum requirements for growing kittens [17]. This assumes that the bioavailability of amino acids in the foods is adequate. Since healthy in-contact horses and cats also had low plasma SAA levels, this anomaly cannot simply be a consequence of anorexia or of the numerous metabolic disturbances that develop secondarily to the multiple system neuropathy. Instead, we hypothesise that SAA depletion may reflect exposure of FD and in-contact cats to xenobiotics, since SAA are strong nucleophiles that trap, reduce, detoxify and facilitate the excretion of electrophilic compounds and free radicals derived from dietary toxins such as mycotoxins, plant lectins, sesquiterpene lactones and toxic amino acids [10–13]. Previous epidemiological investigations on all three premises [14–16], and of other FD clusters [3,30–31] have failed to identify potential causal xenobiotics with the exception of *Clostridium botulinum* type C neurotoxin [32]. While there is an *association* between *C*. *botulinum* and both FD and EGS [5–7], this does not prove causality, and the authors are unaware of a biologically plausible link between low SAA status and toxic-infection with *C*. *botulinum*. Interestingly, the prolonged occurrence of multiple cases of FD on premise A ceased when the diet previously fed to the FD and in-contact cats was replaced, the environment disinfected and the breeding toms replaced. There were no further occurrences of FD in Premise A following these interventions. While it is unclear which of these interventions was protective, we consider that removal of a dietary xenobiotic, such as a neurotoxic mycotoxin, produced by microbes contaminating the dry pelleted food in the feed hoppers, is most likely.

To test the hypothesis that FD and the associated alterations in plasma amino acid status were attributable to ingestion of mycotoxins in the dry food, multi-mycotoxin analyses were performed on dry food from all 3 premises. All samples contained beauvericin, deoxynivalenol, enniatins (A1, B and B1), fumonisin (B1, B2 and B3), fusarenon X and zearalenone, while deoxynivalenol-3-glucoside and mycophenolic acid were also detected in food from 2 premises. Consistent with these data, previous studies have identified mycotoxins in a high proportion of pet food samples, with the mycotoxin profiles and levels being comparable to those reported herein [33–36]. The mycotoxin profile identified in all foods was consistent with Fusarium *spp*. contamination [37,38]. Fungal contamination may have occurred post-manufacture or may reflect fungal contamination of raw ingredients such as cereals, since deoxynivalenol is stable during extrusion processing of cat feed [39].

We consider it unlikely that the any of mycotoxins detected caused FD. Firstly, all detected mycotoxins were present only at low levels, well below the guidance limits for cat foods (limits are available only for T2, HT2 toxin and ochratoxin A) and for other pet foods [39–44]. Secondly, only deoxynivalenol and the fumonisins have reported neurotoxic effects *in vivo* [42,45–47]. While some of the clinical signs of FD could theoretically be attributed to the actions of one of these neurotoxic mycotoxins; for example, profound anorexia and swallowing deficits have been associated with deoxynivalenol ingestion in other species [45,46], the reported clinico-pathological consequences of their ingestion differ from those associated with FD [2,3]. Further work is required to test our hypothesis that FD is caused by another neurotoxic mycotoxin or xenobiotic present in the dry food which was not detected using the multi-mycotoxin screen.

We hypothesise that depletion of SAA in FD reflects glutathione-mediated hepatic detoxification of mycotoxins or other xenobiotics. Since glutathione contains cystine, a derivative of methionine, this process may deplete SAA, while methionine supplementation can attenuate the detrimental effects of mycotoxins [10–13,48].

Since methionine is required for protein synthesis, as a methyl group donor, and part of the coenzyme (*S*-adenosylmethionine) for methyl group transfer, methionine deficiency results in a multitude of metabolic aberrations [20]. Whether low SAA status contributes to the neurodegeneration in FD and EGS is unknown. However, methionine deficiency cannot be the sole cause of FD since removal of methionine from the diet of kittens and adult cats caused weight loss, lethargy, and excessive ocular secretions [24,49,50] but did not produce signs suggestive of FD. Consequently we hypothesise that FD is caused by the direct neurotoxic effects of a dietary mycotoxin or xenobiotic rather than by the associated depletion of methionine.

The majority of cats had glutamate concentrations exceeding the reference range (50–100 μmol/L [20]). This probably reflects artefactual contamination of plasma/sera with glutamate-rich platelets [51], rather than a real effect on glutamate metabolism. Consistent with this possibility, equine plasma had excessively high glutamate concentrations unless harvested by ultracentrifugation [9]. Arginine, which is essential for cats, was limiting in 4 FD, 2 in-contact and 2 control cats. However, in-contact and control cats showed no signs of arginine deficiency, which include anorexia, severe weight loss, diarrhoea and hyperammonemia [52,53]. While tyrosine concentrations were limiting in 5 FD, 2 in-contact and 2 control cats, these cats had no signs of tyrosine deficiency, which include weight loss, hair pigment change, hyperactivity, excessive vocalisation, hypersalivation and ataxia [54,55].

## Conclusions

The reduction in plasma/serum concentrations of methionine in FD and in-contact cats is consistent with the reduction in plasma SAA (cysteine and methionine) concentrations in horses with acute EGS. Food analysis indicated that the low methionine status in FD and in-contact cats is unlikely to be attributable to dietary inadequacy of methionine and/or cystine. Several mycotoxins were detected at low levels in dry food from 3 premises, however none of these appears to induce the specific clinico-pathologic features which characterise FD. Further work is required to test our hypothesis that FD, and the low SAA status of FD cats, EGS horses and in-contact animals, reflects exposure to an unidentified neurotoxic mycotoxin or xenobiotic.

## Supporting information

S1 Table The 72 mycotoxins tested for and their respective reporting limits (μg/kg). (PDF)

S2 Table Concentrations (μmol/L) of amino acids in serum/plasma from individual cats with feline dysautonomia (FD; n = 14), in contact cats (IC; n = 5) and control cats (CONT; n = 6). (PDF)
