# Supplementary material for: Alterations in amino acid status in cats with feline dysautonomia
Source: PLoS One. 2017 Mar 23;12(3):e0174346. doi: 10.1371/journal.pone.0174346 (PMC5363954; doi:10.1371/journal.pone.0174346)
Supplement: S1 Table — (PDF) [file pone.0174346.s001.pdf]

Supplementary List 1; The mycotoxins tested for in the multi-mycotoxin screen, and their respective reporting limits* (µg/kg).

Compound	Max. Reporting Limit / µg/kg
3-Acetyl-deoxynivalenol	50
15-Acetyl-deoxynivalenol	50
Aflatoxin B1	2.5
Aflatoxin B2	2.5
Aflatoxin G1	2.5
Aflatoxin G2	2.5
Aflatoxin M1	2.5
Aflatoxin M2	2.5
Altenuene	50
Alternariol	50
Alternariol monomethyl ether	50
Beauvericin	5
Citrinin	50
Cyclopiazonic acid	50
Cytochalasin A	50
Cytochalasin B	50
Cytochalasin D	50
Cytochalasin E	50
Cytochalasin H	50
Deepoxy-deoxynivalenol	50
Deoxynivalenol	50
Deoxynivalenol-3-glucoside	50
Diacetoxyscirpenol	50
Emodin	50
Enniatin A	5
Enniatin A1	5
Enniatin B	5
Enniatin B1	5
Ergocornine	25
Ergocorninine	5
Ergocristine	25
Ergocristinine	5
Ergocryptine	25
Ergocryptinine	5
Ergometrine	25
Ergometrinine	5
Ergosine	25
Ergosinine	5
Ergotamine	25
Ergotaminine	5

Compound	Max. Reporting Limit / µg/kg
Fumonisin B1	50
Fumonisin B2	50
Fumonisin B3	50
Fusarenon X	50
Fusaric acid	50
Gliotoxin	250
HT2 toxin	50
Meleagrin	50
Moniliformin	50
Mycophenolic acid	50
Neosolaniol	50
3-Nitropropionic acid	500
Nivalenol	100
Ochratoxin A	25
Patulin	250
Penicillic acid	50
Penitrem A	50
Phomopsin A	50
Roquefortine C	5
Sterigmatocystin	2.5
T2 toxin	50
Tentoxin	50
Tenuazonic Acid	50
Verruculogen	50
Wortmannin	50
α-Zearalanol	25
β-Zearalanol	25
Zearalanone	25
α-Zearalenol	25
β-Zearalenol	25
Zearalenone	25
Zearalenone-4-sulphate	50

*Reporting limits are half the concentration of a Level of Interest that was determined during method development, based on a number of factors including legislative levels, analyte toxicity and analyte response (sensitivity). These Reporting limits are equivalent to the lowest concentration analytical standard run during the method (equal to 0.5 x the Level of Interest). In some cases this is the lowest level that can be measured with good precision and signal to noise, but for many cases the instrument sensitivity means that much lower levels can be determined. In all in this table these are described as maximum reporting limits but for many compounds it is possible to measure at lower concentrations, although for accurate quantification in this range additional analytical standards would need to be included. The method was intended to be used as a screening method and levels reported are indicative.
