# Supplementary material for: Alterations in amino acid status in cats with feline dysautonomia
Source: PLoS One. 2017 Mar 23;12(3):e0174346. doi: 10.1371/journal.pone.0174346 (PMC5363954; doi:10.1371/journal.pone.0174346)
Supplement: S2 Table — (PDF) [file pone.0174346.s002.pdf]

Supplementary Table 1; Concentrations (umol/L) of amino acids in serum/plasma from individual cats with feline dysautonomia (FD; n=14), in contact cats (n=5) and control cats (n=6). Also shown are plasma amino acid concentrations (umol/L) for kittens fed diets lacing that amino acid [AA(-)], for kittens fed diets containing each amino acid at minimum requirements (MR) and for kittens fed diets co

	AA(-)	MR	AI	FD 1	FD 2	FD 3	FD 4	FD 5	FD 6	FD 7	FD 8	FD 9	FD 10	FD 11	FD 12	FD 13	FD 14	IC 1	IC 2	IC 3	IC 4	IC 5	CONT 1	CONT 2	CONT 3	CONT 4	CONT 5	CONT 6
**Alanine**				249	460	255	683	417	457	894	392	371	790	442	369	486	543	465	399	725	945	957	295	694	278	497	474	575
**Arginine**	*28*	*75*	*100*	34	246	129	211	184	158	55	155	26	36	87	124	141	187	101	54	59	182	167	111	87	68	108	115	50
**Aspartate**				19	23	20	97	39	35	52	35	38	65	48	31	67	31	62	116	92	86	114	71	68	89	33	48	61
**Cyst(e)ine**				<10	<5	<5	10	15	15	15	<10	13	19	11	12	11	<10	<10	<10	22	15	18	<10	<10	<10	<10	<10	<10
**Glutamate**	*50-100*		*50-100*	70	46	63	179	116	92	123	86	78	104	131	104	124	78	298	532	478	477	515	19	88	108	109	124	112
**Glutamine**				486	912	381	719	358	371	509	408	532	492	381	346	608	619	550	464	266	437	267	729	819	729	913	866	1323
**GLU + GLU**				556	958	444	898	474	463	632	494	610	596	512	450	732	697	848	996	744	914	782	748	907	837	1012	990	1435
**Glycine**				254	784	304	388	777	498	902	304	357	586	354	407	673	241	556	487	465	626	349	660	880	618	360	421	594
**Histidine**	*9*	*55*	*100*	110	169	115	127	100	89	68	62	104	142	59	66	139	121	101	143	124	69	84	85	71	92	129	117	167
**Isoleucine**	*8*	*30*	*75*	128	117	137	115	70	90	116	92	102	53	90	62	94	111	40	48	73	87	75	31	24	59	49	44	31
**Leucine**	*25*	*75*	*125*	184	230	193	198	153	175	221	154	160	144	133	134	204	185	85	138	214	234	163	83	53	136	156	121	94
**Lysine**	*45*	*60*	*110*	163	237	169	294	191	236	321	211	234	140	220	153	186	157	138	62	64	117	125	115	97	91	87	120	64
**Methionine**	*11*	*30 (70)* [1]	*45*	55	68	55	33	58	46	68	45	49	39	42	43	90	49	11	52	64	11	<10	65	69	122	90	77	77
**Ornithine**				170	58	34	24	51	67	258	48	144	181	101	70	136	38	51	36	53	33	35	31	39	29	35	36	32
**Phenylalanine**	*11*	*25 (75)* [2]	*65*	75	67	61	100	67	94	88	74	74	63	72	58	69	107	39	53	60	55	41	57	49	80	55	65	71
**Serine**				169	372	151	282	284	210	404	158	201	362	145	256	432	179	280	250	307	352	236	193	255	200	239	237	650
**Taurine**				250	369	324	185	364	489	360	221	176	125	187	166	74	67	116	211	125	276	273	128	128	179	60	101	91
**Threonine**	*60*	*80*	*150*	183	221	153	242	152	186	201	140	161	158	126	128	231	294	133	134	219	202	157	111	109	139	155	130	105
**Tyrosine**	*10*	*35*	*50*	51	56	50	58	28	37	48	31	33	42	41	31	31	75	28	25	63	61	28	50	27	59	41	42	28
**Valine**	*33*	*66*	*130*	276	341	261	369	242	342	363	290	284	232	263	225	358	290	151	183	128	131	253	100	98	159	141	151	83

[1] **without cystine in the diet**

[2] **without tyrosine in the diet**

ntaining each amino acid at ≥150% minimum requirements (AI).
